# Large spin-orbit coupling and helical spin textures in 2D heterostructure [Pb_2_BiS_3_][AuTe_2_]

**DOI:** 10.1038/srep35313

**Published:** 2016-10-12

**Authors:** L. Fang, J. Im, W. DeGottardi, Y. Jia, A. Glatz, K. A. Matveev, W.-K. Kwok, G. W. Crabtree, M. G. Kanatzidis

**Affiliations:** 1 Materials Science Division, Argonne National Laboratory, Lemont, IL 60439, USA; 2 Advanced Material Division, Korea Research Institute of Chemical Technology, Daejeon 305-600, Korea; 3 Chemistry Department, Northwestern University, Evanston, IL 60208, USA; 4 NUFAB, Northwestern University, Evanston, IL 60208, USA; 5 Department of Physics, Northern Illinois University, DeKalb, Illinois 60115, USA

## Abstract

Two-dimensional heterostructures with strong spin-orbit coupling have direct relevance to topological quantum materials and potential applications in spin-orbitronics. In this work, we report on novel quantum phenomena in [Pb_2_BiS_3_][AuTe_2_], a new 2D strong spin-orbit coupling heterostructure system. Transport measurements reveal the spin-related carrier scattering is at odds with the Abrikosov-Gorkov model due to strong spin-orbit coupling. This is consistent with our band structure calculations which reveal a large spin-orbit coupling gap of ε_so_ = 0.21 eV. The band structure is also characterized by helical-like spin textures which are mainly induced by strong spin-orbit coupling and the inversion symmetry breaking in the heterostructure system.

The motion of an electron through a lattice potential gives rise to a coupling between the electron’s momentum and its spin. This relativistic effect is known as spin-orbit coupling (SOC). SOC plays an important role in topologically nontrivial electronic structures^1^,^2^,^3^ as well as those which can realize Majorana fermions^4^,^5^,^6^. SOC also offers a unique route to tune the spin degree of freedom that has potential impact on spintronics devices^7^,^8^,^9^. Understanding SOC in confined systems such as interfacial boundaries and nanowires is particularly important in order to exert quantum control on electronic degrees of freedom in miniaturized devices. Extensive studies have been conducted in the 2D electron gas such as GaAs/Ga_1-x_Al_x_As^10^ and HgTe/CdTe/HgTe quantum wells^11^. More emergent properties can be expected in similar systems with sufficiently strong SOC such as naturally formed crystalline heterostructures that adopt a structure similar to those of epitaxially grown heterostructure films. A strong advantage of naturally formed heterostructures is that a large number of organic and inorganic materials adopt this type of structure, for example the intergrowth chalcogenides^12^ and the inorganic-organic hybrid systems such as halides^13^. These crystal heterostructures provide a parallel platform to epitaxial films to uncover novel quantum phenomena derived from strong SOC and inversion symmetry breaking^10^,^11^.

Recent work on the naturally formed 2D mineral [Pb_2_BiS_3_][AuTe_2_] shows heterostructure electronic states featuring a conductive [AuTe_2_] layer sandwiched between two insulating [Pb_2_BiS_3_] sheets^14^. The building blocks [Pb_2_BiS_3_]^+1^ and [AuTe_2_]^−1^ are alternately stacked along the crystal’s *c*-axis to form an intergrowth structure, shown in Fig. 1a 14,15,16. A number of intriguing properties such as an extremely large electrical anisotropy, high mobility 2D carriers, and linear energy band dispersions were discovered^14^. The SOC strength in this material is expected to be strong, because many of the component materials are heavy elements. However, as we will discuss below, the SOC in [Pb_2_BiS_3_][AuTe_2_] is anomalous in that it lies beyond the expected scaling properties predicted by the Abrikosov-Gorkov model^9^. Furthermore, our calculations on atomically thin films unveil helical-like spin textures when the spin vectors traverse around the closed 2D Fermi surfaces. The helical-like spin textures could be an effect arising from the strong SOC and the unique heterostructure in [Pb_2_BiS_3_][AuTe_2_].

## Results and Discussion

A direct measurement of a materials’ SOC strength is difficult because SOC is a relativistic effect. However, SOC manifests itself in quantum interference phenomena such as weak antilocalization (WAL)^17^,^18^. WAL provides an effective approach to quantitatively investigate the SOC strength of a material using standard electrical characterization. In the diffusion regime and in the case of self-intersecting electron scattering paths, the interference between the electron’s time reversed paths is pronounced and tends to localize electrons. This phenomenon is known as weak localization (WL). WAL occurs when strong SOC suppresses WL effects.

**Figure 1:**
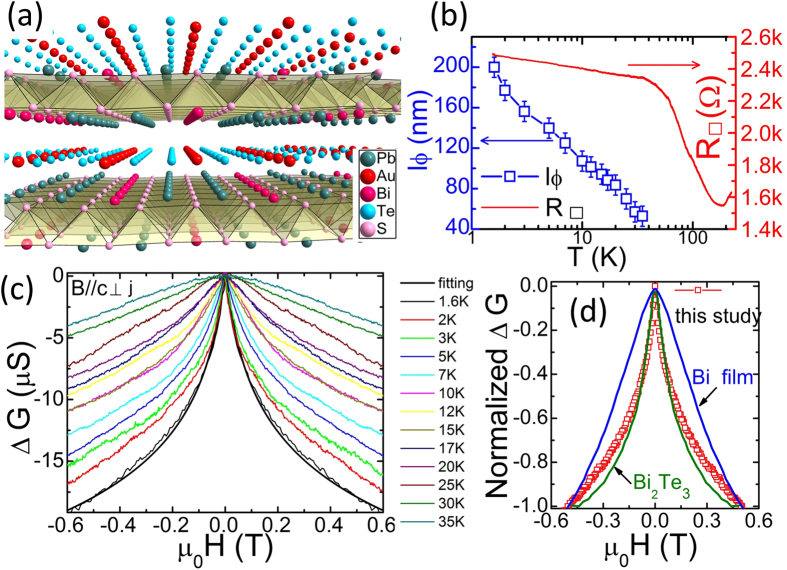
(**a**) The intergrowth structure of [Pb_2_BiS_3_][AuTe_2_] is constituted by two building blocks [Pb_2_BiS_3_]^+^ and [AuTe_2_]^−^, which stack alternatively along the *c*-axis. (**b**) Temperature dependent resistance and phase coherence length of a thin sheet crystal [Pb_2_BiS_3_][AuTe_2_]. (**c**) The field dependent conductance at elevated temperatures. The cusp shape curve at 1.6 K can be fitted with the 2D Hikami-Larkin-Nagaoka equation. (**d**) WAL of [Pb_2_BiS_3_][AuTe_2_] resembles that of Bi and TI Bi_2_Te_3_. The temperature is 2 K.

Figure 1b shows the temperature dependent resistance of a cleaved single-crystal thin layer. The resistance upturn below 150 K demonstrates the poor metallic property of the samples. Below 40 K, the resistance increases linearly with decreasing temperature. This behavior could be related to electron-electron interaction that has been extensively discussed in disordered semiconductors and topological insulators^19^,^20^. Our magnetoconductivity measurements focus on the low field regime, for example B < 0.6 T. In this regime, the Hall resistivity (ρ_xy_) is far smaller than the longitudinal resistivity (ρ_xx_) and the square conductance tensor takes the form 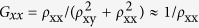. The magnetoconductance is defined as 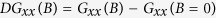. Figure 1c is the field dependent conductivity at different temperatures. Both its cusp shape and its negative sign are hallmarks of WAL. Due to thermal fluctuations, the cusp shape gradually broadens with increasing temperature and diminishes at 40 K.

We apply the 2D Hikami-Larkin-Nagaoka (HLN) equation^21^,^22^ to fit the data. Assuming that τ_*φ*_ is much longer than both τ_so_ and τ_e_, the HLN equation [see equation (2)] can be written


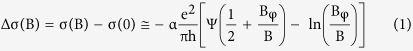


where α = 1/2, e is the electron charge and h is the Planck constant. The parameters τ_e_, τ_so_, and τ_*φ*_ are the electronic elastic scatting time, spin-orbit scattering time and phase coherence time, respectively. The quantity *Ψ(z)* is the digamma function. We define a field strength *B*_*φ*_  = *ħ*/(4*e*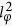) which is the characteristic field associated with the coherence length *l*_*φ*_, where 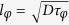 and *D* is the diffusion constant. Figure 1c shows a fitting curve which virtually superposes on the experimental data. This fitting confirms that [Pb_2_BiS_3_][AuTe_2_] has strong SOC. At elevated temperatures (i. e. T = 35 K), *l*_*φ*_ = 50 ± 10 nm, indicating robust quantum interference against thermal fluctuation in this system. Moreover, the agreement with a theoretical model of 2D transport confirms that the electronic degrees of freedom are confined to 2D in [Pb_2_BiS_3_][AuTe_2_]. Previous studies have shown large electrical anisotropy of *Г*~10^4^ in this material^14^.

In Fig. 1d, we compare conductance data as a function of magnetic field of [Pb_2_BiS_3_][AuTe_2_] with that of Bi_2_Te_3_^23^ and Bi^24^ at T = 2 K. For purposes of comparison, this data has been normalized to unity (see supporting information). The similarities among these various systems, especially between [Pb_2_BiS_3_][AuTe_2_] and Bi_2_Te_3_, is surprising. The topological insulator (TI) Bi_2_Te_3_ and semi-metal Bi are known for having unusually strong SOC among materials systems. This striking resemblance indicates that aspects of the SOC coupling in [Pb_2_BiS_3_][AuTe_2_] may be unusual. In order to gain a deeper understanding, we quantitatively investigated τ_so_ and τ_e_ of [Pb_2_BiS_3_][AuTe_2_] by analyzing the HLN equation that contains a form describing different scattering processes^17^,^21^,^22^.


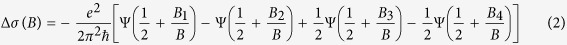


where *B*_1_ = *B*_e_ + *B*_so_ + *B*_*s*_, *B*_2_ = 4/3*B*_so_ + *B*_*φ*_ + 2/3*B*_*s*_, *B*_3_ = *B*_*φ*_ + 2*B*_*s*_, *B*_4_ = *B*_2._ The characteristic fields *B*_n,n_ = _1–4_ are connected with the characteristic relaxation times τ_n_ by the relation *B*_n_ = ħ/4e*D*τ_n_. The parameter *B*_*s*_is a characteristic field due to magnetic scattering and is zero in this case because [Pb_2_BiS_3_][AuTe_2_] is not a magnetic material with no magnetic impurities. It is worthy pointing out that Bergmann^17^ uses a special case of the formula derived by Hikami, Larkin, and Nagaoka^21^. Specifically, the corresponding decoherence times are the same for all three components of the spin. In addition, there is a typo in the HLN equation which has been corrected by Maekawa and Fukuyama^22^. Fitting data to equation (2) is challenging due to the large parameter space of *B*_e_, *B*_SO_ and *B*_*φ*_. This problem can be addressed by using the value of *B*_*φ*_ that was determined using equation (1), which assumes that τ_*φ*_ ≪ τ_so_, τ_e_ still holds at low temperatures. Our fitting yields *B*_so_ = 0.5 ± 0.2 T and *B*_e_ = 1 ± 0.2 T. The reported *B*_so_ of Bi is 0.25 T^24^. The reported value of *B*_so_ of Bi_2_Te_3_ is greater than 8 T^25^. In contrast, the *B*_so_ of material systems with moderate SOC are generally far less than 0.1 T (see supporting information). The large *B*_so_ for these three systems qualitatively explains their similar WAL data. Our fitting parameters are reasonable: a value of *B*_e_ = 1 ± 0.2 T corresponds to a mean free path of ~10 nm which is consistent with the sample’s poor metallic properties as revealed by the resistivity measurement presented in Fig. 1b.

**Figure 2:**
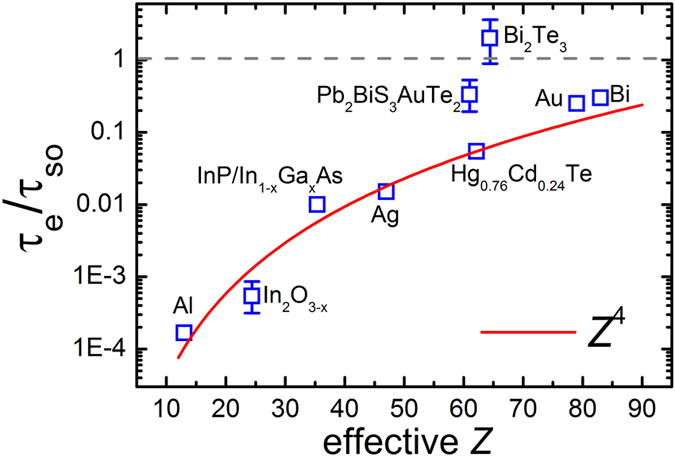
τe/τs_o_ versus effective atomic number (*Z*) for different systems. Weighted arithmetic mean is used to calculate the effective atomic number of materials composed of multiple elements. For [Pb_2_BiS_3_][AuTe_2_], only the elements Au and Te are accounted for the *Z* because the density of states at the Fermi surface are mainly contributed by Au *3d* electrons and Te *p* electrons^14^. The red solid line is a simulation using the relation *Z*^4^. TI Bi_2_Te_3_ violates the Abrikosov and Gorkov’s prediction τ_e_/τ_so_~ *Z*^4^ due to the topology-related Berry’s phase and the extremely large SOC. [Pb_2_BiS_3_][AuTe_2_] does not follow the *Z*^4^ relation. The τ_so_ and τ_e_ of Al, InP/InGaAs, In_2_O_3_, Hg_0.76_Cd_0.24_Te, Au and Ag are from references^28^,^35^,^36^,^37^,^38^.

Materials with large SOC strength may have potential applications as platforms for spin-orbitronics^7^. Comparison of the SOC related carrier scattering in various systems is thus worthwhile. Here we use the model of Abrikosov and Gorkov which predicts τ_e_/τ_so_ ≈ (α*Z*)^4^,^9^ where α ≈ 1/137 is the fine structure constant and *Z* represents the atomic number. This model reveals that the electrons’ spin-orbit scattering is not only determined by the atomic number, but also is influenced by the elastic scattering of electrons by impurities. This is particularly important for spin-orbitronics applications since it predicts that τ_so_ can be tuned by the material’s purity. Here, we use the weighted arithmetic mean to calculate the effective atomic number of materials composed of multiple elements. For [Pb_2_BiS_3_][AuTe_2_], only the elements Au and Te are accounted for in the effective *Z* since the density of states at the Fermi surface are mainly contributed by Au *3d* electrons and Te *p* electrons^14^. Figure 2 summarizes the relations between τ_e_/τ_so_ and the effective atomic mass of a variety of systems whose SOC strength has been investigated. Consistent with the Abrikosov-Gorkov prediction, a majority of the materials follow the trend that τ_e_/τ_so_ is proportional to *Z*^4^, which is depicted by the red solid line in Fig. 2. The topological insulator Bi_2_Te_3_ is an exception due to its extremely large SOC^26^, the topology-related Berry’s phase and the backscattering immunity^27^ which always make τ_so_ extremely short and τ_e_ exceptionally long. In topological insulators, the elastic scattering rates can be weaker than the spin orbit scattering rates^8^,^25^, which results in τ_e_/τ_so_ being greater than unity. The topological insulators are thus considered to be excellent spin-generator materials^7^. We categorize the TI material Bi_2_Te_3_ into the unconventional class of systems and the rest such as Au^28^ into the conventional class. Our [Pb_2_BiS_3_][AuTe_2_] sample falls between these two classes, suggesting the strong SOC, heterostructured systems may have potential for spin generation. The violation of the Abrikosov-Gorkov scattering model in our system is intriguing, and must be related to strong SOC.

**Figure 3:**
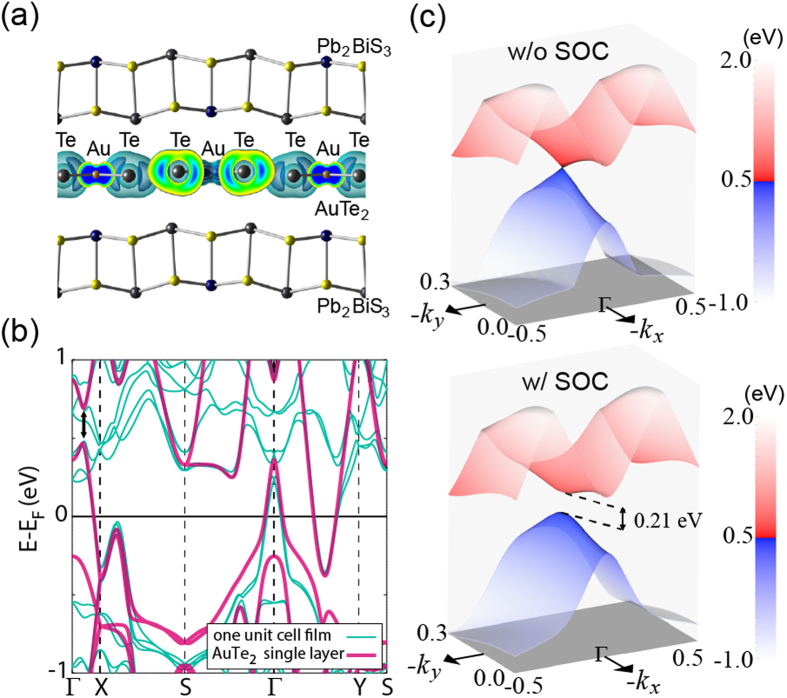
(**a**) Contour plot of the local density of state (LDOS) of [Pb_2_BiS_3_][AuTe_2_] near the Fermi level. The [AuTe_2_]^−^ layer dominates the electronic structure. (**b**) Energy bands dispersion of one-unit-cell [Pb_2_BiS_3_][AuTe_2_] film (light blue), and [AuTe_2_]^−^ single layer (light red). A hole pocket lies at Г and an electron pocket is formed between Г-Y. (**c**) A Dirac-like gapless state appears at the hole-band without SOC (upper panel). SOC opens the Dirac-like gapless state with a spin-orbit gap of 0.21 eV (lower panel). The gap opening is denoted in (**b**) by the black arrow.

The large SOC can be understood by our *ab initio* calculations. The electronic structure near the Fermi level of [Pb_2_BiS_3_][AuTe_2_] purely originates from the [AuTe_2_]^−^ layer. Figure 3a is a contour plot of the local density of state (LDOS) of [Pb_2_BiS_3_][AuTe_2_] near the Fermi level, which clearly shows that the electronic states are tightly confined to the [AuTe_2_]^−^ layer. Figure 3b exhibits the energy band dispersions of the [AuTe_2_]^−^ layer and the single-unit-thick films. The vertical arrow marks the gap opening due to strong SOC. Figure 3c shows the detailed information of the gap opening in 3D reciprocal space. Without SOC in the [AuTe_2_]^−^ layer, a Dirac-like gapless state appears at the hole-band between the Г and X points. SOC opens the gapless Dirac-like state to form a spin-orbit gap of ε_SO_ = 0.21 eV. This value is comparable to the SOC-induced band inversion energy in TIs and the spin-orbit gap of the topology-nontrivial Sb-bilayer system^26^,^29^. The SOC gap in the [AuTe_2_]^−^ layer also occurs at the Г point near the Fermi Energy (see the top panel of Figure S6 in supporting information). The large spin-orbit gap elucidates the large SOC and the violation of the Abrikosov-Gorkov scattering model. Since [Pb_2_BiS_3_][AuTe_2_] is not a TI, our studies thus suggest that materials can violate the Abrikosov-Gorkov scattering model as long as their SOC strengths are sufficiently large.

**Figure 4:**
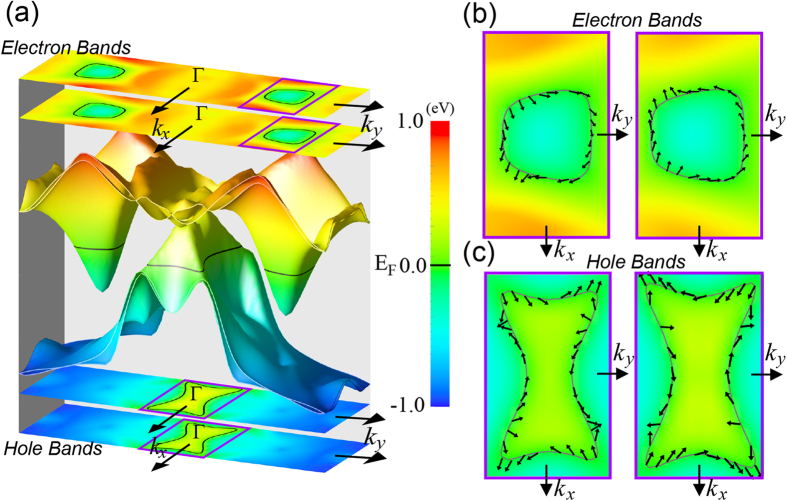
(**a**) A 3D plot of the electronic band structure of the one-unit-cell [Pb_2_BiS_3_][AuTe_2_] film near the Fermi level. The 2D projection of electron bands and hole bands are depicted on the top and at the bottom, respectively. Black solid lines indicate the Fermi level. The dual projected sheets are due to inversion symmetry breaking on the surfaces of the one-unit-cell film. (**b,c**) shows spin direction along the closed 2D Fermi surfaces for electron and hole bands, respectively. Left and right panels of (**b,c**) correspond to two non-degenerated bands due to inversion symmetry breaking in the one-unit-cell film. The area of (**b,c**) in momentum space are denoted by purple-colored rectangles shown in (**a**).

The large SOC, combined with the unique heterostructure in [Pb_2_BiS_3_][AuTe_2_], may lead to helical spin textures that have been observed in topological insulators and semiconductor heterostructure films^27^,^30^. We carried out theoretical investigations on the single-unit-cell film [Pb_2_BiS_3_][AuTe_2_] films where the twofold spin-degeneracy is lifted due to inversion symmetry breaking. Figure 4a presents the Fermi surfaces of the electron and hole bands and their projections at the Fermi level. The dual projection sheets are induced by broken inversion symmetry. Both electron bands and hole bands are found to have spin structures whose direction changes as a function of crystal momentum *k*. The spin textures of the electron and hole pockets exhibit different topological properties. For the electron pockets, the spin does not undergo a full winding as one traverses around the Fermi surface while for the hole pockets the spin winds around 3 times. The helical-like spin texture in the hole pocket can be attributed to the strong SOC and the inversion-asymmetry induced spin splitting in a heterostructure system. For example the Ga_1-x_In_x_As/InP quantum well has shown helical spin texture in the presence of SOC^30^. The spin textures in the hole pocket exhibit spin-flip between the hole carriers with opposite momentum *k* and –*k*, as a consequence of time-reversal symmetry. The shape of the Fermi surface reflects the orthorhombic crystalline symmetry of [Pb_2_BiS_3_][AuTe_2_]. In addition, the spin-flip in the hole pocket may indicate a non-vanishing Berry’s phase. Direct detection of the Berry’s phase is beyond the scope of this study. However, the WAL in our sample is consistent with a nontrivial Berry’s phase which gives opposite signs to time-reversed electron paths. This type of destructive quantum interference can also result in WAL^18^,^27^,^31^. Our modeled helical spin textures in single-unit-cell films could be experimentally pursued as we demonstrate that single-unit-cell films naturally occur on the cleaved surfaces of a bulk crystal (Figure S1 in supporting information). Further experiments such as spin-resolved photoemission spectroscopy are needed to confirm our theoretical predictions of the helical spin textures.

## Conclusions

To summarize, large SOC strength was discovered in the 2D heterostructure [Pb_2_BiS_3_][AuTe_2_]. This large SOC is induced by a large spin-orbit gap ε_SO_ = 0.21 eV. More broadly, our work suggests that materials can violate the Abrikosov-Gorkov scattering model for sufficiently strong spin-orbit coupling. *Ab initio* calculations reveal helical-like spin textures and spin-flips at the Fermi surfaces. These predictions can be attributed to the effect of strong SOC and inversion symmetry breaking in the heterostructure as well as the constraint of crystalline symmetry in the AuTe_2_ layer. More generally, our work points out that naturally forming heterostructures made of heavy atoms provide a new direction for exploring novel quantum phenomena at the atomic scale. Given the large number of naturally formed organic and inorganic heterostructures and their hybrids^12^,^13^, more discoveries can be expected in this direction.

## Methods

Bulk crystal synthesis follows a self-flux method by melting stoichiometric compositions of [Pb_2_BiS_3_][AuTe_2_]. Details of the crystal synthesis were reported in our previous paper^14^. Crystals were exfoliated on SiO_2_/Si wafers using the so-called scotch tape method and were post-annealed at 350 °C in argon gas to remove chemical residuals on the cleaved surface. The crystals’ thickness is determined by AFM (PSIA Corporation). Morphology studies on cleaved surfaces were conducted by a MultiMode scanning probe microscope (Veeco) operated in a peak-force tapping mode that has vertical spatial resolution up to 50 pm. Cleaved crystals with thickness 40 nm were selected for transport characterization. Standard photolithography (LaserWirter, MICROTECH) and magnetron sputtering were employed to pattern contacts on cleaved crystals. In-plane electrical characterization was conducted using the standard four probe method in a liquid ^4^He variable temperature cryostat equipped with a triple-axis vector magnet system (AMI).

Electronic structures of [Pb_2_BiS_3_][AuTe_2_] bulk, one-unit-cell thick [Pb_2_BiS_3_][AuTe_2_] film and the [AuTe_2_]^−^ single layer were calculated within density functional theory using Projector Augmented Wave method^32^ implemented in the VASP code^33^. Perdew-Burke-Ernzerhof type^34^ generalized gradient approximation was utilized for the exchange correlation functional. Spin-orbit coupling was included in the non-collinear form. Experimentally resolved crystal structure was employed for [Pb_2_BiS_3_][AuTe_2_], and crystal structure of [AuTe_2_]^−^ single layer was prepared from the bulk crystal structure of [Pb_2_BiS_3_][AuTe_2_] by removing [Pb_2_BiS_3_]^+^ layers. The crystal structure of the monolayer [Pb_2_BiS_3_][AuTe_2_] is calculated assuming a vacuum layer of 3 nm.

## Additional Information

**How to cite this article**: Fang, L. *et al*. Large spin-orbit coupling and helical spin textures in 2D heterostructure [Pb_2_BiS_3_][AuTe_2_]. *Sci. Rep.* **6**, 35313; doi: 10.1038/srep35313 (2016).

## Supplementary Material

Supplementary Information
