# Supplementary material for: Large spin-orbit coupling and helical spin textures in 2D heterostructure [Pb2BiS3][AuTe2]
Source: Sci Rep. 2016 Oct 12;6:35313. doi: 10.1038/srep35313 (PMC5059675; doi:10.1038/srep35313)
Supplement: Supplementary Information [file srep35313-s1.pdf]

Supporting Information

Large spin-orbit coupling and helical spin textures in 2D heterostructure [Pb_2BiS_3][$AuTe_2$]

L. Fang[1,3], J. Im[2], W. DeGottardi[1], Y. Jia[4], A. Glatz[1,5], K. Matveev[1], , W.-K. Kwok[1], G. W. Crabtree[1], M. G. Kanatzidis[1,3]

[1]Materials Science Division, Argonne National Laboratory, Lemont, IL 60439
[2]Advanced Material Division, Korea Research Institute of Chemical Technology, Daejeon 305-600, Korea
[3]Chemistry Department, Northwestern University, Evanston, IL 60208
[4]NUFAB, Northwestern University, Evanston, IL 60208
[5]Department of Physics, Northern Illinois University, DeKalb, Illinois 60115, USA

I. The cleaved surface morphology of [Pb_2BiS_3][$AuTe_2$]

The *c*-axis length of [Pb_2BiS_3][$AuTe_2$] is 9.34Å. Previous structure determinations [S1-S3] have shown that weak chemical bonds Pb(Bi)-Te connects the building blocks of [Pb_2BiS_3] and [$AuTe_2$]. Sample cleavage breaks the interlayer bonds and leaves either the [Pb_2BiS_3] layer or the [$AuTe_2$] layer exposed to the ambient environment with equal probability. AFM measurements were conducted to investigate the surfaces morphology, shown in (c) and (d). The AFM equipped with a peak-force tapping mode has a vertical resolution up to 50 pm. (e) summarizes the depth information of the cleaved surfaces shown in (d). 80% of the surface is 1.08 nm deep and the rest is 1.5 nm deep. Comparing with the structure of [Pb_2BiS_3][$AuTe_2$], we conclude that the 1.08 nm deep surfaces are one-unit-cell thick films and the 1.5 nm thick surfaces correspond to one and half unit cell. It is worth mentioning that the cleaved surfaces are stable in ambient environment although surface oxidation could happen.

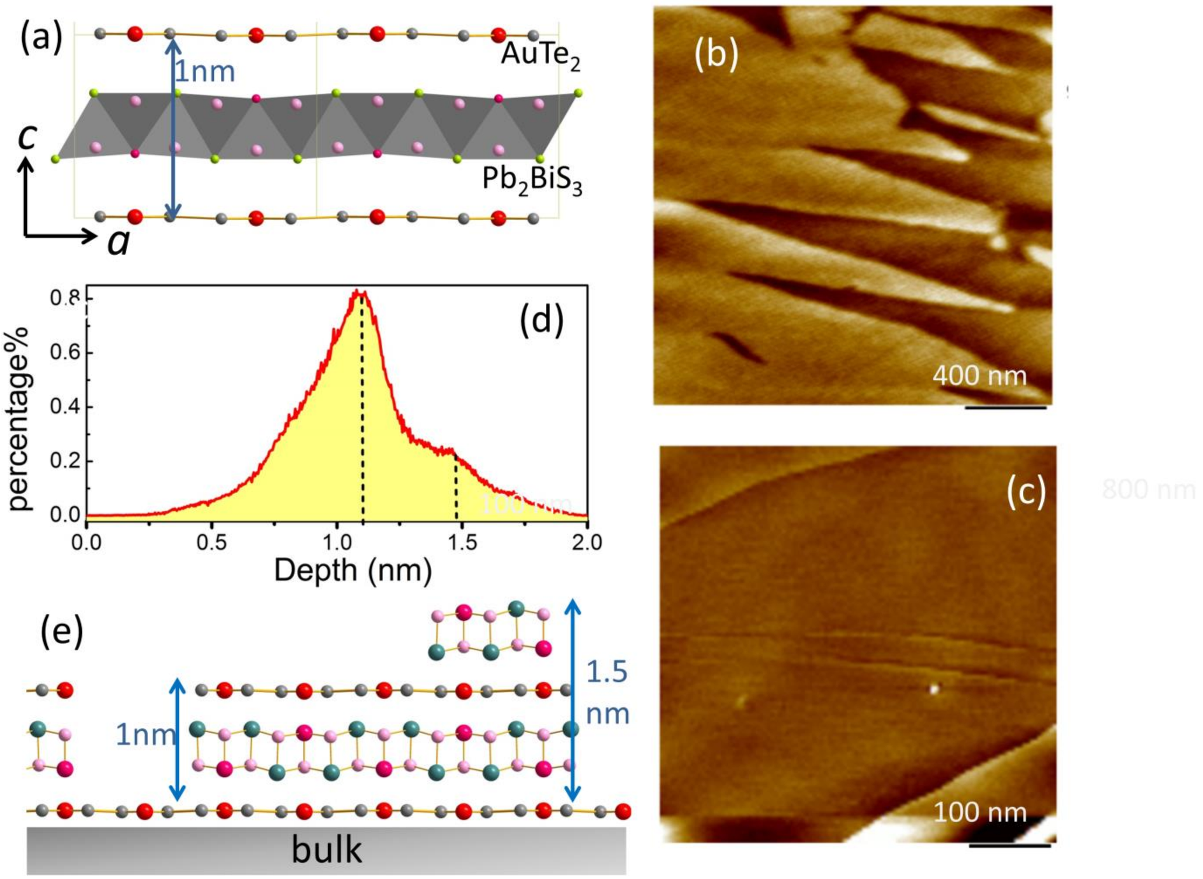

Fig. S1. (a) The intergrowth structure of $[Pb_2BiS_3][AuTe_2]$ consists of two building blocks, $[Pb_2BiS_3]$ and $[AuTe_2]$, which stack alternatively along the *c*-axis. (b)-(c) are high resolution AFM images of the cleaved surfaces of a single crystal $[Pb_2BiS_3][AuTe_2]$. (d) summarizes the depth information of the same area that is shown in (b). 80% of the surface is 1.08 nm deep and the rest is 1.5 nm deep. The unit of the vertical axis is relative. (e) is a schematic picture showing the depth of the cleaved surfaces that was determined by AFM.

II. Weak antilocalization and spin-orbit scattering fields of different systems

For systems with small effective atomic numbers such as GaAs [S4] and silver [S5], the cusp-like WAL curves disappear quickly with increasing magnetic field. For example, WAL of silver only occurs in small fields between ±0.02 T. By contrast, heavy element Bismuth [S6] and TIs [S7-S8] show more pronounced WAL effects. The SOC characteristic fields B_{so} of different systems increase with increasing (effective) atomic numbers with an exception for the TIs. The strong SOC and the Berry's phase in TIs lead to a remarkably large B_{so}, for example B_{so} >8T has been reported in reference S9.

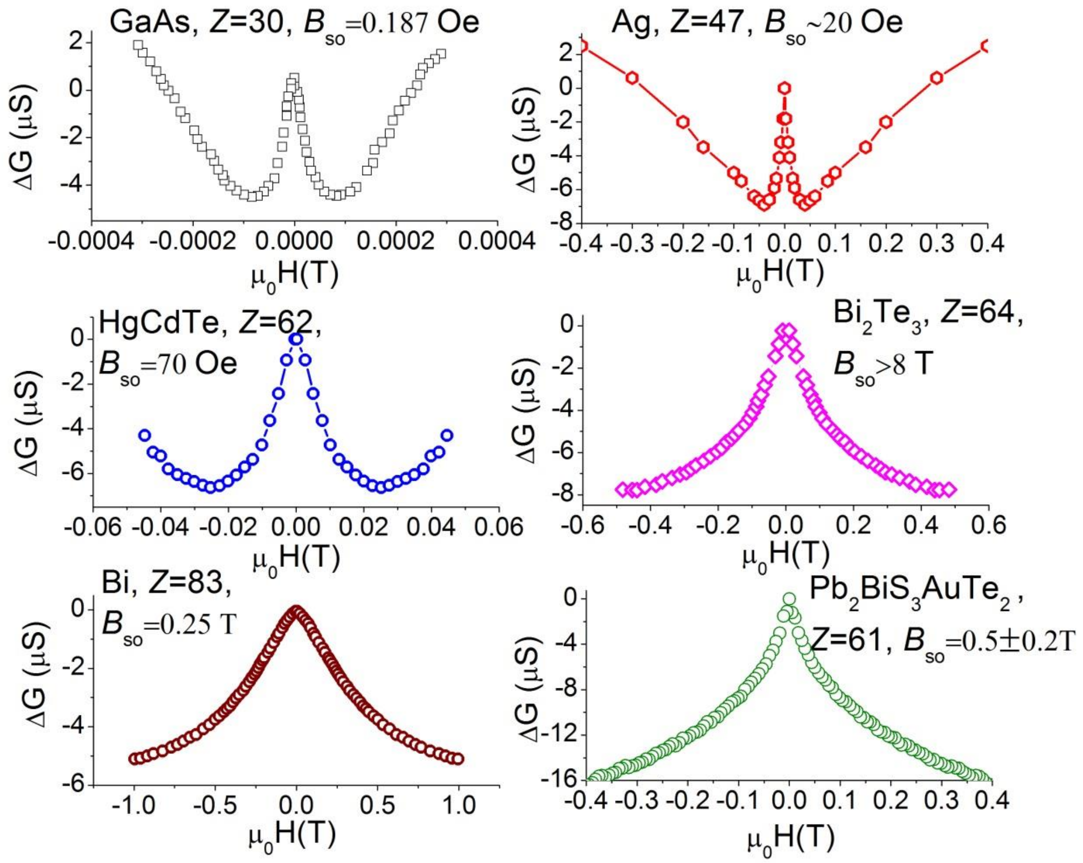

Fig. S2. WAL of different systems at temperatures 1.6-2K.

III. Qualitative analysis of the WAL

For 2D, non-magnetic and strong SOC systems, fitting WAL using the HLN equation (Eq.2) requires three unknown parameters B_e, B_{so} and B_φ, which are the characteristic fields of elastic scattering, spin-orbit scattering and phase coherence, respectively. Three fitting parameters normally can generate large uncertainties. To bear this in mind, fitting parameters need to be well consistent with experimental results and known properties of materials. We focus on the WAL data at 1.6 K because that τ_φ much longer than both τ_{so} and τ_e is still satisfied at this temperature. Using Eq. 1, the best fit leads to B_φ=0.004 T, corresponding to a phase coherence length $l\varphi$≈200 nm. The 200 nm long $l\varphi$ is reasonable because that Bi_2Te_3 demonstrates a similar WAL curvature with that of [Pb_2BiS_3][$AuTe_2$] and Bi_2Te_3 possesses a similar $l\varphi$≈300 nm at 2 K. Fig. S3 shows the fitting. By setting B_φ=0.004 ±0.001 T, Eq. 2 can simulate the data using B_e=1±0.2 T and B_{so}=0.5±0.2 T. Fig. S4 demonstrates the best fitting using Eq. 2. The relatively large B_e=1±0.2 T corresponds to a short mean free path less than 10nm, which is consistent with the poor metallic behavior (R_square≈2000Ω) in our samples. The fitted B_{so}=0.5±0.2 T can be justified by comparison to the reported results in other strong spin-orbit coupling systems. For example, the spin-orbit scattering time τ_{SO} are 0.01ps and 0.015 ps for our samples and the element Bi, respectively [S6].

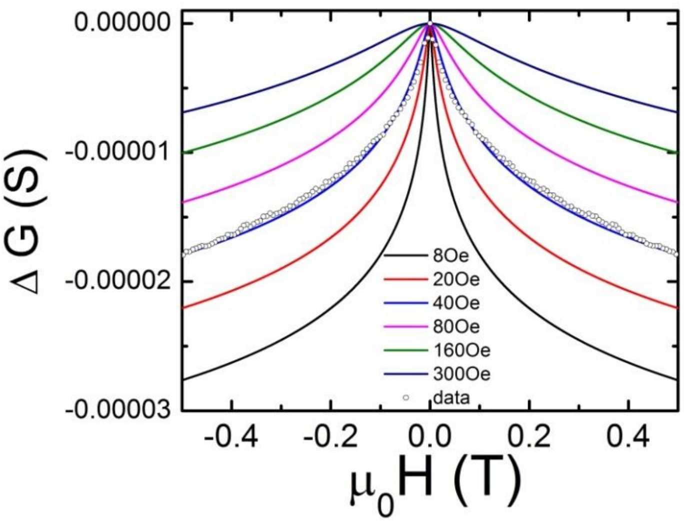

Fig. S3. The WAL data at 1.6 K can be fitted by the HLN equation (Eq.1) with fitting parameter B_φ≈40 Oe.

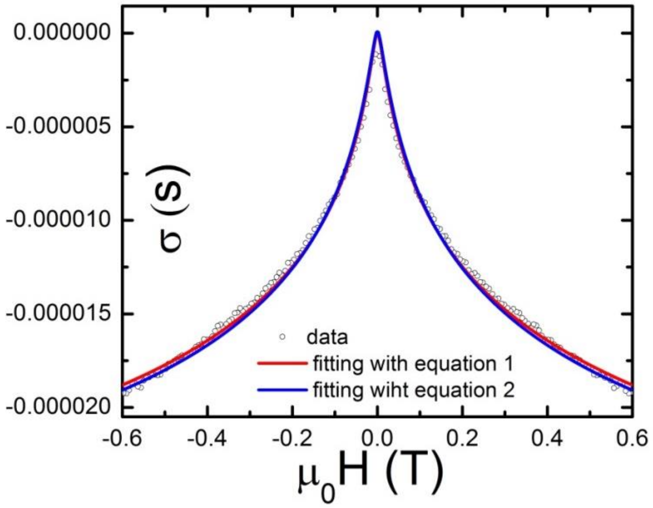

Fig. S4. The WAL data at 1.6 K can be fitted by the HLN equation (Eq.2) with fitting parameters B_φ=0.004 ±0.001 T, B_e=1±0.2 T and B_{so}=0.5±0.2 T. In applied magnetic fields -0.6 T≤μ_0H≤0.6 T, Eq. 2 is an excellent approximation to Eq. 1 and no significant improvement in the fitting is obtained by using Eq. 2.

IV. Two-dimensionality WAL in [Pb_2BiS_3][$AuTe_2$]

The 2D characteristic can be determined not only by the field dependence, but also by the temperature dependent phase coherence length $l\varphi$. It is known that, for electron-electron scattering in two-dimensional systems, the inelastic scattering diffusion length as a function of temperature follows a power-law dependence T^{-n} with exponent n≈1/2 [S10]. Assuming the inelastic scattering diffusion length is approximately equal to the phase coherence length $l\varphi$ [S11], $l\varphi$ should also follow the $T^{-1/2}$ relation. Below is the experimental data of the temperature dependent $l\varphi$ of [Pb_2BiS_3][$AuTe_2$] (left panel). The data can be fitted by $T^{-1/2}$, suggesting the 2D behavior of WAL. Moreover, the 2D electronic structure of [Pb_2BiS_3][$AuTe_2$] determines the dimensionality of the transport behavior. For example, the Fermi Surfaces of [Pb_2BiS_3][$AuTe_2$] are cylinder-like (right panel).

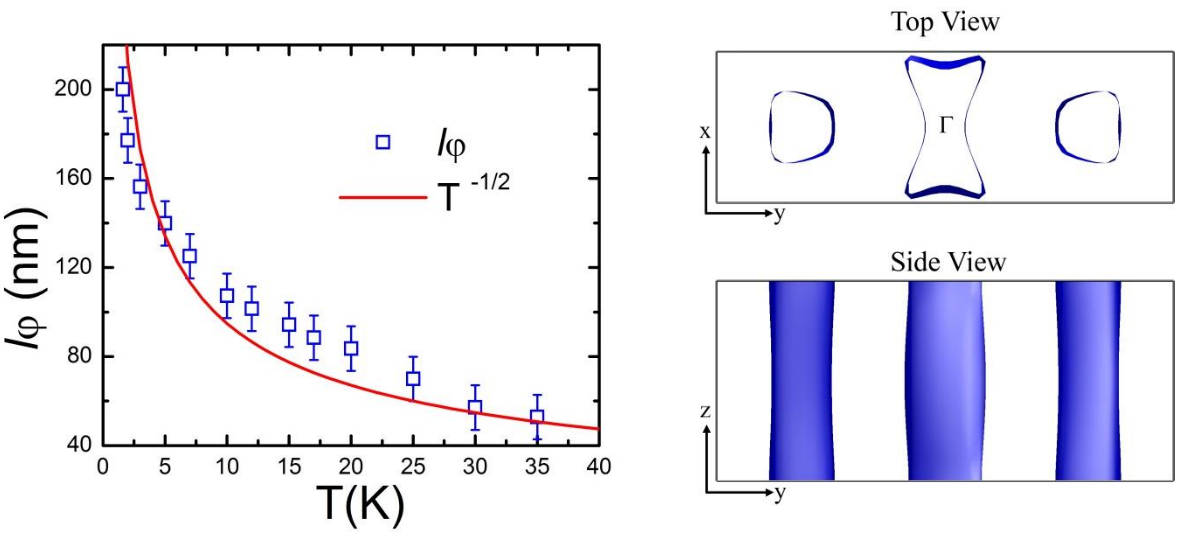

Fig. S5. (left panel) The temperature dependent phase coherence length *l*φ shows a 2D characteristic as the data can be fitted by a 2D electron-electron scattering model (red curve). (right panel) The Fermi Surfaces of $[Pb_2BiS_3][AuTe_2]$ are cylinder-like, suggesting a 2D electronic structure.

V. Spin-orbit gap in bulk and one-unit-cell film $[Pb_2BiS_3][AuTe_2]$

Unlike the electronic structure of $[AuTe_2]^-$ single layer, gap opening of the Dirac-like band is not clearly shown in the bulk and the one-unit-cell $[Pb_2BiS_3][AuTe_2]$ film because of the coexistence of $[Pb_2BiS_3]$ bands in the gap by accident. Here, by projection to $[AuTe_2]^-$ layer, we present the same spin-orbit gap in the bulk and the one-unit-cell film $[Pb_2BiS_3][AuTe_2]$. Figure S6 shows layer-projected band structure of the $[AuTe_2]^-$ single layer in (a-b), one-unit-cell $[Pb_2BiS_3][AuTe_2]$ film in (c-d), and $[Pb_2BiS_3][AuTe_2]$ bulk in (e-f). Compared to the case of bulk $[Pb_2BiS_3][AuTe_2]$, the electronic states from the $[Pb_2BiS_3]^{+1}$ layer are in lower energy state in the one-unit-cell film. This can be attributed to an additional dipole moment along the z-direction by breaking inversion symmetry.

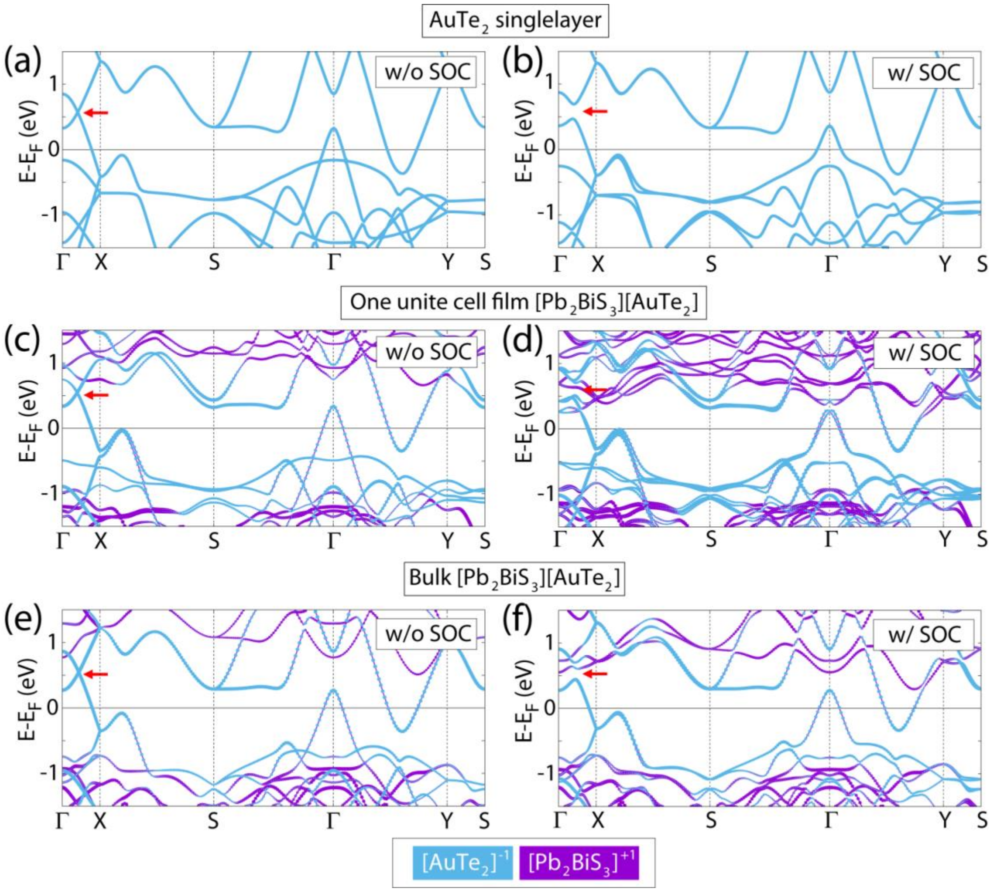

Fig. S6. Layer-projected band structure of $[AuTe_2]^-$ single layer, one unit cell thick $[Pb_2BiS_3][AuTe_2]$ film and the $[Pb_2BiS_3][AuTe_2]$ bulk in (a-b), (c-d) and (e-f), respectively. Red arrows assist to show the location of Dirac-like gapless bands without SOC and the gap opening with SOC. The light blue curves correspond to the projection to $[AuTe_2]^-$ layer and the purple curves correspond to the $[Pb_2BiS_3]^+$ layer.

References

[S1] Francis, C. A., Criddle, A. J., Stanley, C. J., Lange, D. E., Shieh, S., Francis, J. G. Buckhornite $AuPb_2BiTe_2S_3$, a new mineral from Boulder County. *Can. Mineral.* **30**, 1039−1047 (1992).

[S2] Effenberger, H., Culetto, F. J., Tapa, D., Paar, W. H. The crystal structure of synthetic buckhornite, $[Pb_2BiS_3][AuTe_2]$. *Z. Kristallogr.* **215**, 10−16 (2000).

[S3] Fang, L; Im, J.; Stoumpos, C. C.; Shi, F.; Dravid, V.; Leroux, M.; Freeman, A. J.; Kwok, W.-K.; Chung, D. Y.; Kanatzidis, M. Two-Dimensional Mineral $[Pb_2BiS_3][AuTe_2]$: High-Mobility Charge Carriers in Single-Atom-Thick Layers. *J. Am. Chem. Soc.* **137** (6), 2311–2317(2015).

[S4] Dresselhaus, P. D., Papavassiliou, C. M. A., Wheeler, R. G., Sacks, R. N. Observation of spin precession in GaAs inversion layers using antilocalization. *Phys. Rev. Lett.* **68**, 106-109 (1992).

[S5] Kawaguti, T., Fujimori, Y. Magnetoresistance and inelastic scattering time in thin films of silver and gold in weakly localized regime. *J. Phys. Soc. Jpn. 52*, 722-725 (1983).

[S6] Sangiao, S.; Marcano, N.; Fan, J.; Morell´on, L.; Ibarra M. R.; De Teresa, Quantitative analysis of the weak anti-localization effect in ultrathin bismuth films. *EPL* **95,** 37002 (2011).

[S7] He, H.-T.; Wang, G.; Zhang, T.; Sou, I.-K.; Wong, G. K. L.; Wang, J.-N.; Lu, H.-Z.; Shen, S.-Q.; Zhang, F.-C. . Impurity Effect on Weak Antilocalization in the Topological Insulator Bi_2Te_3. *Phys. Rev. Lett.*, **106**, 166805 (2011).

[S8] Hong, J.; Lee, J.; Joo, S.; Rhie, K. Control of the spin-orbit coupling by gate voltage in semiconductor FET structures. *J. Korean Phys. Soc.* **45**, 197-201(2004).

[S9] Zhang, S. X.; McDonald, R. D.; Shekhter, A.; Bi, Z. X.; Li, Y.; Jia, Q. X.; Picraux S. T Magneto-resistance up to 60 Tesla in topological insulator Bi_2Te_3 thin films. *Appl. Phys. Lett.* **101**, 202403 (2012).

[S10] Altshuler, B. L. Effects of electron-electron collisions with small energy transfers on quantum localization. *J. Phys. C: Solid State Phys.***15**, 7367(1982).

[S11] Santhanam, P.; Wind, S.; Prober, D. E. Localization, superconducting fluctuations, and superconductivity in thin films and narrow wires of aluminum. Phys. Rev. B 35, 3188 (1987).
